# Differences in *MHC-B* diversity and KIR epitopes in two populations of wild chimpanzees

**DOI:** 10.1007/s00251-019-01148-3

**Published:** 2019-12-03

**Authors:** Vincent Maibach, Kevin Langergraber, Fabian H. Leendertz, Roman M. Wittig, Linda Vigilant

**Affiliations:** 1 Department of Primatology, Max Planck Institute for Evolutionary Anthropology, Deutscher Platz 6, 04103 Leipzig, Germany; 2 School of Human Evolution and Social Change, Arizona State University, Tempe, AZ 85281 USA; 3 Institute of Human Origins, Arizona State University, Tempe, AZ 85281 USA; 4 Robert Koch-Institute, 13353 Berlin, Germany; 5 Taï Chimpanzee Project, CSRS, Abidjan, 01 Côte d’Ivoire

**Keywords:** MHC, Next-generation sequencing, *Pan troglodytes*, primates

## Abstract

The major histocompatibility complex (MHC) class I genes play a critical role within the immune system, both by the presentation of antigens from intracellular pathogens to immunocompetent cells and by the interaction with killer cell immunoglobulin-like receptors (KIR) on natural killer cells (NK cells). Genes of the MHC are highly diverse, and MHC variation can have effects on the immune functionality of individuals; hence, comparisons of MHC diversity among closely related phylogenetic taxa may give insight into the factors responsible for the shaping of its diversity. The four geographically separated chimpanzee subspecies differ in their overall genetic diversity, have different population histories, and are confronted with different pathogens in their natural habitat, all of which may affect MHC class I DNA sequence diversity. Here, we compare the *MHC-B* exon two DNA sequence diversity from 24 wild western and 46 wild eastern chimpanzees using necropsy and noninvasively collected fecal samples, respectively. We found a higher *MHC-B* exon two nucleotide diversity, in our western than eastern chimpanzees. The inclusion of previously published *MHC-B* exon two data from other western and eastern chimpanzees supported this finding. In addition, our results confirm and extend the finding of a very low C1 epitope frequency at eastern chimpanzee MHC-B molecules, which likely affects the ability of these molecules to interact with NK cells. While the understanding of the differing pathogen environments encountered by disparate populations of a species is a challenging endeavor, these findings highlight the potential for these pathogens to selectively shape immune system variation.

**Electronic supplementary material:**

The online version of this article (10.1007/s00251-019-01148-3) contains supplementary material, which is available to authorized users.

## Introduction

The mammalian immune system is critically dependent on genes of the major histocompatibility complex (MHC), which encodes for cellular surface proteins responsible for the presentation of antigens to immunocompetent cells (Townsend and Bodmer 1989). Two different classes of MHC molecules can be distinguished. MHC class II molecules are involved in the presentation of extracellular antigens and thus defend against extracellular pathogens. In contrast, MHC class I molecules defend against intracellular pathogens by presenting antigens mostly derived from viral proteins or cancer-infected cells (reviewed in Rock et al. 2016; Sommer 2005). Along with their role in antigen presentation to T cells, MHC class I molecules also interact with killer cell immunoglobulin-like receptors (KIR) occurring on natural killer cells (NK cells) (Parham 2005; Parham et al. 2010). NK cell responses are the result of a balance of stimulating and inhibiting signals derived from cell surface receptors like KIRs (Lanier 2003). In the case of viral infection, cells can lose expression of MHC class I molecules and thereby the ability to present antigens to T cells as well as the ability to interact with KIRs inhibiting NK cell activation, making such infected cells targets for NK cell lysis (Hoglund and Brodin 2010). Thus, the NK cell responses substantially support the defense against viral infections by contributing to the innate immunity (Waggoner et al. 2016; Wroblewski et al. 2019).

Relative to protein-coding genes in the rest of the genome, MHC class I genes display an extreme degree of polymorphism. Thousands of alleles have been described for the three classical MHC class I genes in humans, designated as *HLA-A*, *HLA-B,* and *HLA-C*, with the *B* locus having the largest number of described alleles (Robinson et al. 2015). Different mechanisms have been proposed to explain the extraordinarily high diversity at MHC loci, mainly focusing on pathogen-mediated selection (Bernatchez and Landry 2003; Doherty and Zinkernagel 1975; Jeffery and Bangham 2000; Spurgin and Richardson 2010). This is the idea that a high diversity of alleles is a response to the high diversity of pathogens typically encountered and that changes in pathogen exposure may drive changes in allele frequencies in populations due to differential survival of individuals.

Comparison of variation at the MHC for humans and our closest living relatives, the African apes, has revealed remarkable differences in lineage and allelic diversity that are thought to be linked to selective sweeps in the past. For example, both the bonobo (*Pan paniscus*) and chimpanzee (*Pan troglodytes*) lack the *A2* lineage of *MHC-A* and exhibit lower diversity in MHC class I introns, which are suggested to be result from a selective event driven by lentivirus exposure some 2 million years ago (Ma), before these congeners speciated (Adams et al. 2000; de Groot et al. 2000; de Groot et al. 2002; Lawlor et al. 1995; McAdam et al. 1995). There is further evidence of functional differences in *MHC-B* in bonobos compared to chimpanzees, which are attributed to selective processes and/or a severe bottleneck in ancestral bonobos since the divergence of these two taxa (Maibach and Vigilant 2019; Wroblewski et al. 2017). The *MHC-B* locus is of particular interest because of its role in HIV disease progression in humans (Carrington and O’Brien 2003; Fellay et al. 2007; Goulder et al. 1996; Kiepiela et al. 2004; Matzaraki et al. 2017; Naranbhai and Carrington 2017) and simian immunodeficiency virus (SIVcpz) infection in chimpanzees (Wroblewski et al. 2015).

Genetic analysis of the four geographically defined chimpanzee subspecies *P. t. verus* (western chimpanzees), *P. t. ellioti* (Nigerian-Cameroon chimpanzees), *P. t. troglodytes* (central chimpanzees), and *P. t. schweinfurthii* (eastern chimpanzees) has shown that they have different degrees of genetic diversity, and experienced different demographic histories (Becquet et al. 2007; Fischer et al. 2006; Fischer et al. 2004; Hey 2010; Prado-Martinez et al. 2013; Wegmann and Excoffier 2010). The western subspecies was the first to diverge from the ancestral population, approximately 500,000 years ago, and the most recent split was between the eastern and central subspecies (Becquet et al. 2007; Caswell et al. 2008; Hey 2010; Prado-Martinez et al. 2013; Wegmann and Excoffier 2010). The long history of subspecies separations, and potentially differing pathogenic environments in their habitats, could have led to different levels of MHC diversity and allele compositions in the subspecies.

Indeed, a specific example of how pathogen pressure may differ among populations is provided by the distribution of SIVcpz, a virus related to HIV which can also cause AIDS-like symptoms and increase mortality in infected apes (Barbian et al. 2017; Keele et al. 2009; Sharp and Hahn 2011). SIVcpz has been detected in the wild only in central and eastern chimpanzees (Heuverswyn et al. 2007; Keele et al. 2006; Prince et al. 2002; Rudicell et al. 2010; Santiago et al. 2003; Santiago et al. 2002; Sharp and Hahn 2011; Sharp et al. 2005; Worobey et al. 2004). Evidence that SIVcpz can influence MHC diversity and allele composition of a population or community comes from a study investigating SIVcpz prevalence in three neighboring eastern chimpanzee communities, which detected changes in the *MHC-B* allele composition of a community over a 15-year period (Wroblewski et al. 2015). Although apparently not subject to infection by SIVcpz, several neighboring communities of western chimpanzees have experienced Ebola virus outbreaks and anthrax infections, pathogens to date not reported in eastern chimpanzees, providing further potential for differential exposure to pathogens to shape immune responses (Formenty et al. 1999; Hoffmann et al. 2017; Leendertz et al. 2004; Leendertz et al. 2017).

In chimpanzees, three different KIR epitopes have been described within the α_1_ protein domain of the exon two of MHC-B and -C molecules (Parham and Moffett 2013; Wroblewski et al. 2015; Wroblewski et al. 2019). MHC-B molecules may have none, the Bw4 or the C1 (MHC-B-C1) epitope, whereby MHC-C molecules have either the C1 (MHC-C-C1) or the C2 epitope (Parham and Moffett 2013; Wroblewski et al. 2015; Wroblewski et al. 2019). Those KIR epitopes interact with KIRs on NK cells, which then depending on the receptor activate or inhibit the NK cell response (Parham 2005).

Studies comparing MHC diversity among chimpanzees sampled at a subspecies or local scale have revealed interesting differences in frequencies of KIR epitopes. For example, although a comparison between a set of captive western and a set of wild-born central chimpanzees resident in an African sanctuary found no differences in nucleotide diversity for the three MHC class I genes *A*, *B* and *C*, the set of western chimpanzees had higher frequencies of MHC-B molecules carrying the Bw4 KIR epitope and of MHC-C molecules carrying the C1 (MHC-C-C1) KIR epitope (Maibach et al. 2017; Wroblewski et al. 2019). In addition, another set of captive western chimpanzees had higher frequencies of MHC-B molecules carrying the Bw4 and the C1 (MHC-B-C1) KIR epitope as compared to the KIR epitopes found on MHC-B molecules of wild eastern chimpanzees from Gombe National Park (Wroblewski et al. 2015). The lower frequencies of MHC molecules with KIR epitopes in central and eastern chimpanzees were suggested to be the result of selection differences among the different subspecies (Wroblewski et al. 2015; Wroblewski et al. 2019). Hence, these findings emphasize the need for additional studies comparing MHC between communities, where variation in local selection caused by pathogens may occur.

Here we compare the *MHC-B* exon two variation using samples of western and eastern chimpanzees from two different National Parks in West and East Africa. Chimpanzee communities in both the Taï National Park (Côte d’Ivoire) and Kibale National Park (Uganda) are known to have experienced several epidemics of respiratory viruses (Emery Thompson et al. 2018; Hoffmann et al. 2017; Köndgen et al. 2008; Negrey et al. 2019; Scully et al. 2018). In addition, western chimpanzees from the Taï National Park were also confronted with outbreaks of Ebola (Formenty et al. 1999; Leendertz et al. 2017). A comparison of different wild chimpanzee populations suggested that the Taï chimpanzee population might exhibit lower survival rates relative to chimpanzees from elsewhere, including Kibale National Park (Hill et al. 2001; Muller and Wrangham 2014; Wood et al. 2017), leading to the possibility of differential selection on MHC diversity.

In this study, we characterize *MHC-B* exon two diversity in geographically localized samples of western and eastern chimpanzees, aiming to confirm and extend previous findings on the distribution of MHC variation in wild chimpanzees. Using DNAs derived from either fecal or necropsy samples, we first compare levels of exon two nucleotide and amino acid diversity of our two sets of samples. Next, we investigate their frequencies of the Bw4 and C1 (MHC-B-C1) KIR epitopes. To put our results into perspective, we include comparisons with published data from different populations of both wild and captive western, central, and eastern chimpanzees.

## Methods

### Samples

We analyzed 28 and 50 individuals representing *P. t. verus* (western chimpanzees) and *P. t. schweinfurthii* (eastern chimpanzees), respectively (Tables 1 and 2). As part of a project on determining causes of death in wild chimpanzees (Hoffmann et al. 2017; Leendertz et al. 2004), necropsy samples are routinely collected at the Taï National Park, Côte d’Ivoire and analyzed at the Robert Koch Institute, Germany (https://www.leendertz-lab.org/). Aliquots of those DNA samples were used in this study to analyze the western chimpanzee *MHC-B* exon two diversity. The 28 western chimpanzees from this study were members of four habituated and one semi-habituated neighboring chimpanzee groups (Table 1) (Herbinger et al. 2001; Kouakou et al. 2011; Wittig 2018). Our sampling of western chimpanzees contains five individuals known to be related (one mother with three offspring with different fathers and two individuals with the same father) (Table 1).

**Table 1:** Necropsy samples from the Taï National Park representing our set of western chimpanzees. Allele 1 and allele 2 show the *MHC-B* exon two sequences of the particular individuals. The two KIR epitopes Bw4 and C1 (MHC-B-C1) at the particular MHC-B molecules are indicated. The absence of a KIR epitopes is shown by dashes (–). Unk. indicates unknown cause of death. Superscript numbers show relatedness of individuals: ^1^Ophelia with her three children, ^2^Ophelia and Shogun have the same father

Individual	Community	Cause of death	Date of sampling	Allele 1	Allele 2	KIR
Dorry	North	Unk.	October 21, 2001	B-12	B-25	C1/C1
Unknown I	North East	Intergroup encounter	July 21, 2015	B-09	B-12	C1/C1
Kady	Middle	Unk.	December 11, 2001	B-08	B-09	–/C1
Noah	Middle	Unk.	February 13, 2002	B-11	B-11	Bw4/Bw4
Leo	Middle	Anthrax	February 14, 2002	B-08	B-10	–/Bw4
Candy	East	Pneumonia	February 07, 2006	B-21	B-12	Bw4/C1
Vasco	East	Pneumonia	February 09, 2006	B-08	B-08	–/–
Porthos	East	Anthrax	April 07, 2008	B-10	B-11	Bw4/Bw4
Dartagnon	East	Anthrax	April 19, 2009	B-09	B-26	C1/C1
Iome	East	Anthrax	April 22, 2009	B-09	B-11	C1/Bw4
Ouganda	East	Anthrax	August 22, 2011	B-08	B-08	–/–
Mila	East	Unk.	January 15, 2012	B-10	B-12	Bw4/C1
Ehra	East	Anthrax	January 08, 2014	B-09	B-28	C1/C1
Tita	South	Anthrax	September 26, 2000	B-08	B-12	–/C1
Olduvai^1^	South	Anthrax	June 13, 2002	B-13	B-11	Bw4/Bw4
Orest^1^	South	Pneumonia	March 10, 2004	B-11	B-13	Bw4/Bw4
Ophelia^1,2^	South	Pneumonia	March 10, 2004	B-14	B-11	Bw4/Bw4
Virunga	South	Leopard/pneumonia	March 19, 2004	B-09	B-08	C1/–
Ishas Baby	South	Unk.	February 10, 2006	B-09	B-11	C1/Bw4
Olivia^1^	South	Leopard	December 07, 2009	B-11	B-11	Bw4/Bw4
Louise	South	Pneumonia	December 07, 2009	B-11	B-12	Bw4/C1
Atra	South	Pneumonia	December 08, 2009	B-11	B-13	Bw4/Bw4
Wapi	South	Pneumonia	December 17, 2009	B-27	B-08	C1/–
Shogun^2^	South	Anthrax	March 07, 2016	B-15	B-16	Bw4/–

**Table 2:** Fecal samples from the Kibale National Park representing our set of eastern chimpanzees. Allele 1 and allele 2 show the *MHC-B* exon two sequences of the particular individuals. KIR epitopes at the particular MHC-B molecules are indicated. The absence of a KIR epitopes is shown by dashes (–)

Extract	ID	Date of sampling	Allele 1	Allele 2	KIR
N322-4	NS257	November 18, 2014	B-03	B-07	Bw4/Bw4
N322-5	NS299	December 10, 2014	B-05	B-06	–/–
N322-6	NS300	January 09, 2015	B-07	B-07	Bw4/Bw4
N322-7	NS222	January 09, 2015	B-04	B-07	Bw4/Bw4
N323-6	ES19	April 18, 2015	B-06	B-06	–/–
N319-2	NS284	July 27, 2014	B-18	B-07	–/Bw4
N319-4	NS286	July 27, 2014	B-03	B-04	Bw4/Bw4
N319-5	NS281	July 27, 2014	B-05	B-06	–/–
N320-6	NS292	October 06, 2014	B-05	B-07	–/Bw4
N320-8	NS294	October 06, 2014	B-06	B-07	–/Bw4
N412-7	B37	January 11, 2015	B-05	B-18	–/–
N403-9	B41	June 24, 2015	B-07	B-06	Bw4/–
N408-4	KT4	September 18, 2015	B-05	B-17	–/Bw4
N410-5	B59	October 18, 2015	B-06	B-06	–/–
N411-1	B80	October 18, 2015	B-07	B-17	Bw4/Bw4
N412-10	B63	November 16, 2015	B-07	B-07	Bw4/Bw4
N413-1	NS12	November 17, 2015	B-06	B-07	–/Bw4
N414-6	B66	December 22, 2015	B-04	B-19	Bw4/–
N415-10	B38	January 15, 2016	B-07	B-06	Bw4/–
N415-9	B40	January 15, 2016	B-06	B-07	–/Bw4
N416-1	B17	January 16, 2016	B-06	B-07	–/Bw4
N444-10	B31	April 04, 2016	B-07	B-04	Bw4/Bw4
N444-6	B5	April 04, 2016	B-07	B-07	Bw4/Bw4
N444-7	KT7	April 04, 2016	B-07	B-06	Bw4/–
N445-2	B61	April 04, 2016	B-06	B-07	–/Bw4
N445-4	NS264	April 04, 2016	B-06	B-04	–/Bw4
N443-7	B103	May 04, 2016	B-04	B-07	Bw4/Bw4
N446-3	B108	May 04, 2016	B-06	B-07	–/Bw4
N446-5	B54	May 19, 2016	B-06	B-06	–/–
N449-7	B114	July 17, 2016	B-05	B-04	–/Bw4
N326-5	NS307	July 25, 2014	B-06	B-04	–/Bw4
N350-6	NS184	May 01, 2015	B-19	B-07	–/Bw4
N387-1	M93	October 16, 2014	B-06	B-06	–/–
N390-10	NS278	November 04, 2014	B-06	B-07	–/Bw4
N391-9	M58	November 18, 2014	B-06	B-04	–/Bw4
N396-4	M31	January 17, 2015	B-07	B-03	Bw4/Bw4
N426-2	M4	September 28, 2015	B-05	B-03	–/Bw4
N378-6	NS365	May 02, 2015	B-06	B-03	–/Bw4
N451-18	TA1	October 20, 2015	B-20	B-18	Bw4/–
N328-9	NS311	August 25, 2014	B-06	B-03	–/Bw4
N344-6	NS332	April 18, 2015	B-04	B-03	Bw4/Bw4
N374-5	NS362	June 21, 2015	B-06	B-07	–/Bw4
N433-8	NS250	March 21, 2016	B-03	B-07	Bw4/Bw4
N331-4	NS173	October 06, 2014	B-07	B-04	Bw4/Bw4
N331-6	NS149	October 10, 2014	B-06	B-06	–/–
N349-3	NS340	November 24, 2014	B-23	B-06	Bw4/–

For the eastern chimpanzees in our study, we used part of a collection of fecal samples that were noninvasively collected in the Kibale National Park, Uganda between 2011 and 2016 as part of long-term study of the habituated Ngogo community and monitoring of the unhabituated neighboring communities (Granjon et al. 2016; Langergraber et al. 2009; Langergraber et al. 2011; Langergraber et al. 2007; Langergraber et al. 2013). Details on sample preparation are described in White et al. (2019). Briefly, fecal samples were extracted and DNAs were genotyped using a panel of microsatellites to establish individual identities. For quality assessment, the genotyped samples (> 1700) were further analyzed by a qPCR assay and with the fragment analyzer system (Large Fragment Standard Sensitivity Kit; Advanced Analytical) to calculate the percentage of chimpanzee-derived DNA in those fecal extracts relative to DNA derived from other sources such as bacteria and fungi (see White et al. 2019). Based on those screening results, we estimated an “evaluation score,” which incorporates the DNA concentration given by qPCR, the DNA concentration given by the fragment analyzer, the average length of DNA molecules given by the fragment analyzer, and the percentage of chimpanzee-derived DNA into account. We calculated for each of these four measures the average value of all samples tested in White et al. (2019) (> 1700) and divided the individual values of these four measures (e.g., DNA concentration by qPCR) of each sample by the average values. This gave a relative value indicating the sample’s performance for each measure, with relative values larger than one indicating that the sample had a higher value than the average of all samples for this particular measure. We next calculated for each sample the product of relative values of each of the four measures and sorted all samples based on this calculated product. Samples with the highest “evaluation scores” were considered the most promising extracts to use in this study. We excluded DNA extracts which were older than 2015 from the dataset, as preliminary results indicated a reduced PCR performance with such extracts. This reduced the initial dataset from more than 1700 samples to 831 samples. From the remaining dataset, we selected 50 samples with the highest evaluation scores for attempting MHC class I *B* exon two amplification and sequencing. We chose a larger number of eastern chimpanzee samples compared to our set of western chimpanzees because we expected a lower PCR success rate using the fecal extracts than the necropsy samples. We analyzed the usefulness of the evaluation score by checking for correlations of the evaluation score or one of the four individual measurements with poor fecal DNA sample performance in the PCRs. Although microsatellite analysis confirmed that the samples used represent different individuals, we did not know whether some of our samples were closely related. However, we expect the frequency of close relatives to be low given the large geographical area sampled and the low proportion of close relatives in wild animal populations (Csillery et al. 2006).

### PCR amplification and library preparation

We conducted PCRs targeting the complete exon two of the chimpanzee MHC class I *B* gene (Supplementary Table 1). Products had an estimated length of about 464 base pairs. PCRs were set up in a total volume of 25 μl containing 1× Buffer B (Kapa Biosystems, Wilmington, MA, USA), 0.2 mM dNTPs, 0.25 μM of each primer, 0.5 units of Kapa2G Robust HotStart DNA polymerase (Kapa Biosystems, Wilmington, MA, USA), and 2 μl of DNA extract. We performed a two-step PCR protocol with an initial denaturation of 5 min at 95 °C, followed by 40 cycles of 15 s at 95 °C and 20 s at 72 °C, finished by a final elongation at 72 °C for 5 min. PCR products were checked by gel electrophoresis on a 2.5% Agarose TAE gel. If amplification was successful, PCR products were cleaned from PCR reagents and primer dimers with AGENCOURT AMPure XP PCR purification (Beckman Coulter Life Sciences, Indianapolis, IN, USA) following suppliers’ instructions, with a ratio of magnetic beads to DNA of 0.8. Cleaned PCR products were the template for a second PCR used to prepare the samples for sequencing by adding two distinctive indices and Illumina P5 and P7 adapters to each sample. In addition to the DNA sequence necessary to amplify exon two, our primer sequences for the first PCR contained at the 5′ ends adapter sequences which were the target for the primer annealing in the second PCR (Supplementary Table 1). This second PCR (indexing PCR) had a total volume of 25 μl with 1× Phusion Hot Start II High-Fidelity PCR Master Mix (Thermo Fisher, Waltham, MA, USA), 0.5 μM of each primer P5-iPCR and P7-iPCR and 0.6 μl of cleaned PCR product from the first PCR. The cycling protocol for the indexing PCR started with an initial denaturation of 30 s at 98 °C, followed by 20 cycles of 10 s at 98 °C, 20 s at 58 °C, and 20 s at 72 °C, finished by a final elongation for 5 min at 72 °C. PCR products of this second PCR were again checked by gel electrophoresis on a 2.5% TAE agarose gel and cleaned by AGENCOURT AMPure XP PCR purification with a ratio of beads to DNA of 0.6. We analyzed each individual in two replicates, i.e., this entire process was done for each individual in two independent reactions. If one of the two replicates per individual failed or showed rather weak bands on the gel electrophoresis during this process, we repeated the PCR reactions for this particular individual a third time. We analyzed all products using the Fragment Analyzer system (Large Fragment Standard Sensitivity Kit; Advanced Analytical) to estimate the DNA concentration of each library (sample). We observed that not every PCR replicate had the necessary DNA concentration (~ 300 pg) for sequencing, although some replicates which appeared to have insufficient DNA concentration as estimated by the gel electrophoresis showed a suitable DNA concentration on the fragment analyzer. Consequently, we had no PCR replicate for three western and two eastern chimpanzees; only one of the two desired replicates for 14 other individuals (five western and nine eastern chimpanzees); and for seven eastern chimpanzees, three replicates available for sequencing. Based on the DNA concentrations estimated using the fragment analyzer, we mixed all libraries (samples) equimolar into two different pools (*n* = 15 and *n* = 124, respectively), with a final molarity of 10 nM and 5.04 nM, respectively.

### Sequencing and sequence processing

We sequenced both libraries on one lane on an Illumina MiSeq platform (Illumina, San Diego, CA, USA) using 250 base pair (bp) paired-end runs with two 7-bp indices. To increase sequence complexity, an indexed Phi X 174 library was added prior to sequencing. In total, we sequenced *MHC-B* PCR products for 25 western and 48 eastern chimpanzees. Bases were called using the standard Illumina base caller Bustard. We used leeHom to merge overlapping mate pairs and trim adapter sequences (Renaud et al. 2014). Demultiplexing of reads, i.e., the assignment of reads to a sample based on their indices, was done by deML (Renaud et al. 2015). We further processed the data by removing reads with an expected number of mismatches greater than one per 100 bases and reads with a length shorter than 250 base pairs using SAMtools 1.3.1 (Li et al. 2009). From the filtered reads, we then extracted the MHC *B* exon two sequences from each sample with jMHC (Stuglik et al. 2011), using the primer sequences from our first PCR. We filtered the output from jMHC by the minimum read length of 100 base pairs and a minimum variant count of 50, i.e., a certain sequence must have had at least 50 reads to be considered. We then checked the output from jMHC for every sample in BioEdit 7.2.5 (Hall 1999) and extracted the alleles for each sample. We considered the sequences with the highest number of reads as the true alleles of an individual, and all other sequences with lower number of reads were removed. These had a few differences to the accepted sequence, as one would expect if these low frequency sequences arose due to polymerase misincorporation during PCR or incorrect base calling during sequencing. In the majority of cases, the difference in the number of reads between the considered alleles (one to several thousand reads) and sequences with errors (a few hundred reads) was relatively large. However, in five instances, one allele had a high number of reads, whereas the second allele had only a few reads which were in the range of the sequences with errors. Despite that low coverage, the second allele was considered as “real” because it showed several differences to the first, well-supported allele (not only a few polymerase misincorporations) and was also found in the replicate of the individual and in other individuals as well. We excluded one western and two eastern chimpanzee individuals from the dataset, because we had no second PCR replicate for these individuals and the samples showed ambiguous sequencing results, i.e., the read number of amplicons was low and several possible sequences were present for these particular samples. This reduced our sample size to 24 western and 46 eastern chimpanzees, respectively. After all filtering steps, we obtained for those individuals on average 2011 (range 93–15,061) reads per amplicon. Finally, we compared all *MHC-B* exon two sequences among the different individuals and, for differentiation, we designated them numerically. It is important to note that this numerical designation is not identical to the official designation recommended by the IPD-MHC, which is based on full-length *MHC-B* alleles (Maccari et al. 2017). Because *MHC-B* exon two sequences may represent several different full length *MHC-B* alleles, we wanted to avoid confusion and preferred a simple numerical designation rather using the official designated full length *MHC-B* allele names.

### Data analysis and statistic

We calculated the nucleotide diversity (∏) of the exon two sequences with the package pegas 0.9 (Paradis 2010) in R (R Core Team 2015). For statistical analysis of differences in nucleotide diversity of the exon two sequences between western and eastern chimpanzees, we conducted a permutation test with 10,000 permutations (Adams and Anthony 1996). We pooled all individuals and resampled for each permutation individuals independent from their taxon origin into two groups of the same size as the original two groups of western and eastern chimpanzees (24 and 46, respectively). In each permutation, we calculated the absolute difference in nucleotide diversity between the two groups and compared this with the observed absolute difference in nucleotide diversity of the two groups of western and eastern chimpanzees. We calculated the *p* value as the proportion of absolute differences in nucleotide diversity of every permutation being larger or equal than the observed absolute difference in nucleotide diversity of the two groups of western and eastern chimpanzees. However, the difference in samples size numbers between western and eastern chimpanzees could have an influence on the results of the permutation test (Huang et al. 2006). We therefore conducted an additional test, where we resampled from both chimpanzee sets 20 individuals to equalize the sample size number for the two groups. We resampled the chimpanzee subsets one million times and calculated each time the nucleotide diversity. In addition, we compared the exon two nucleotide diversity of our western and eastern chimpanzee sets with the exon two nucleotide diversity of western, central, and eastern chimpanzee sets described in other studies. We accounted for the different number of sampled individuals in those studies by resampling for each chimpanzee set 20 individuals, which was the minimum sample size number in two of those studies from the literature. We resampled 20 individuals of each particular chimpanzee set one million times (including also our western and eastern chimpanzee sets) and calculated each time the nucleotide diversity.

For the analysis of the diversity of the exon two amino acid sequences, we used the protein variability server which calculates the Shannon entropy, the Simpson diversity index, and the Wu-Kabat variability coefficient (Garcia-Boronat et al. 2008). Other statistical tests are indicated at the corresponding section in the results. For the Fisher exact tests, we used the R package rcompanion 1.3.2 (Mangiafico 2015). We accounted for multiple testing by using the Bonferroni correction which relies on the adjustment of the significant threshold by dividing the initial significant threshold of 0.05 by the number of tests conducted on the same dataset. The adjusted significant thresholds are presented with the statistical tests in the results.

## Results

We started our project with samples from 28 and 50 different western and eastern chimpanzees and were able to produce unambiguous sequencing results for 24 and 46 individuals, respectively. Hence, only four necropsy (western chimpanzees) and four fecal (eastern chimpanzees) samples failed. Specifically, the PCR success rate was 80% (45 of 56) using DNAs derived from necropsy samples and 77% (94 of 112) for DNAs derived from feces.

To guide future research using DNA from feces, we checked if one of our four screening measures (DNA concentrations given by qPCR and fragment analyzer, the average length of DNA molecules, and the percentage of chimpanzee-derived DNA) or the composite evaluation score correlated with poor fecal DNA sample performance. We found a weak negative correlation between our evaluation score and the failure of a DNA extract to amplify in one of the PCR replicates, indicating that a low evaluation score slightly increased the probability that a sample failed in one of the PCR replicates (Pearson’s product-moment correlation, *r* = − 0.316, *t* = − 2.308, df = 48, *p* value = 0.025). None of the four individual screening measurements showed a correlation with PCR success rate (data not shown).

**Fig. 1:**
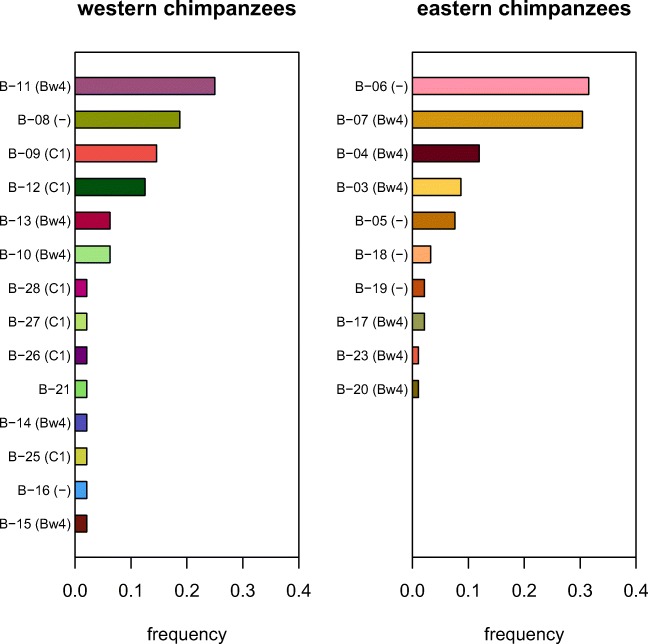
The frequencies of the different *MHC-B* DNA sequences found in our set of western (24 individuals) and eastern chimpanzees (46 individuals) from the Taï National Park and the Kibale National Park, respectively. KIR epitopes present at the particular MHC-B sequences are indicated in brackets. MHC-B sequences have either Bw4, MHC-B-C1, or none (dash) KIR epitope

In total, we found 14 and 10 different nucleotide sequences from the 24 western and 46 eastern chimpanzees, respectively (Tables 1 and 2; GenBank accession numbers: MN213635–MN213661). Two of the 14 western chimpanzee sequences (B-11 and B-21) had an identical exon two DNA sequence but one nucleotide substitution in the intron one region (Table 3). We therefore found 13 *MHC-B* exon two DNA sequences for western and 10 for the eastern subspecies. The number of differences distinguishing the exon two sequences within these two sets ranged from 1 to 33 differences, with an average number of differences of 19.4 for western and 18.2 for eastern chimpanzees. Each of the 13 and 10 different nucleotide sequences coded for a unique amino acid sequence. Comparison with exon two sequences from the literature indicated that three of our 13 western chimpanzee sequences have not been previously described, while all 10 eastern chimpanzee exon two sequences were previously reported (Table 3). None of the exon two sequences were shared between the two subspecies (Fig. 1).

**Table 3:** Comparison of exon two sequences found in this study with complete *MHC-B* sequences from the literature. New alleles are indicated in italics. ^#^Allele B-11 and B-21 had an identical exon two sequence but one nucleotide substitution in the intron one region and are therefore listed together in this table

Exon two	Found in	Equivalent to exon two sequence from these complete *MHC-B* gene sequences
B-03	Eastern	Patr-B*30:01,Patr-B*23:07,Patr-B*30:02,
B-04	Eastern	Patr-B*07:02,Patr-B*07:04,Patr-B*07:05,
B-05	Eastern	Patr-B*38:01,Patr-B*38:02,
B-06	Eastern	Patr-B*22:01,Patr-B*22:03,Patr-B*22:04,Patr-B*22:05,Patr-B*39:01,Patr-B*22:07
B-07	Eastern	Patr-B*33:01:01:01,Patr-B*07:03,Patr-B*33:01:01:02
B-08	Western	Patr-B*02:01,Patr-B*05:01,Patr-B*05:02
B-09	Western	Patr-B*13:01,Patr-B*11:04
B-10	Western	Patr-B*03:01,Patr-B*03:02
B-11/B-21^#^	Western	Patr-B*01:01,Patr-B*09:01,Patr-B*10:01
*B-12*	Western	–
B-13	Western	Patr-B*24:01,Patr-B*24:02
B-14	Western	Patr-B*20:01:01,Patr-B*20:02,Patr-B*20:01:02
*B-15*	Western	–
*B-16*	Western	–
B-17	Eastern	Patr-B*22:02,Patr-B*34:01
B-18	Eastern	Patr-B*17:02
B-19	Eastern	Patr-B*17:03
B-20	Eastern	Patr-B*19:04
B-23	Eastern	Patr-B*23:01:01,Patr-B*23:02,Patr-B*23:05,Patr-B*23:01:02,
B-25	Western	Patr-B*08:02
B-26	Western	Patr-B*16:01:01,Patr-B*16:01:02
B-27	Western	Patr-B*17:01
B-28	Western	Patr-B*04:02

**Fig. 2:**
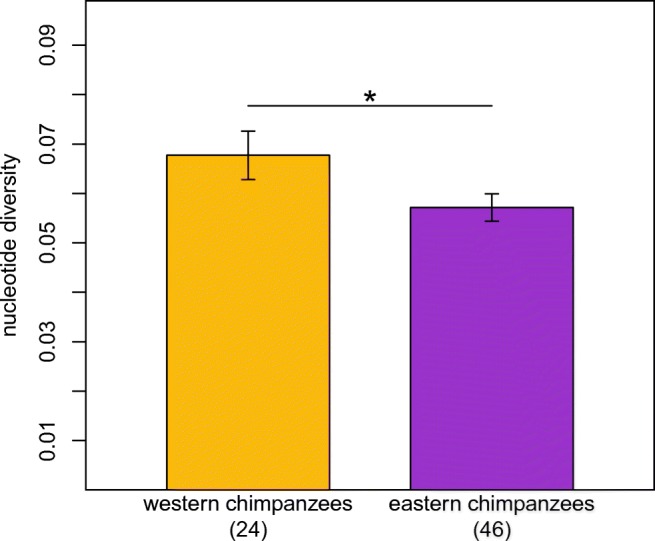
The DNA nucleotide diversity of the *MHC-B* exon two sequences of our sets of western (golden) and eastern chimpanzees (purple). The star above the bars indicates a significant difference between the two sets of sequences (permutation test, *p* = 0.0058). Sample size is indicated below the bars

**Fig. 3:**
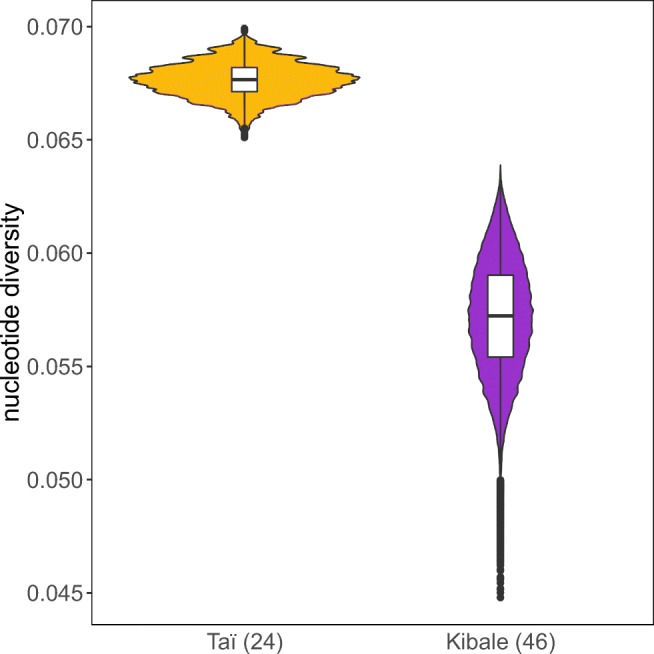
The *MHC-B* exon two nucleotide diversity of our sets of western (golden) and eastern chimpanzees (purple) based on one million resamplings of 20 individuals to match sample size numbers between the two sets of chimpanzees. The shape of the particular plots indicates the distribution of these calculated nucleotide diversities. The number of individuals of the original groups is indicated in brackets

We compared the allele frequencies of the exon two sequences between our samples of the two subspecies. In each of our sampled populations, the allele frequencies were not evenly distributed. We found one (B-11) or two (B-06 and B-07) high-frequency alleles, respectively, as well as alleles with intermediate or low frequencies (Fig. 1). Interestingly, we found fewer *MHC-B* exon two sequences (10) in our set of eastern chimpanzees than in our set of western chimpanzees (13), despite the sample size of eastern chimpanzees being nearly twice that of western chimpanzees. This suggests relatively lower diversity in our set of eastern chimpanzees. To further analyze this observation, we compared the nucleotide diversity of the exon two sequences between western and eastern chimpanzees (Fig. 2). We found a significantly higher nucleotide diversity in our set of western chimpanzees (permutation test, *p* = 0.0058). However, the results of the permutation test could be biased by the differing number of sampled chimpanzees (Huang et al. 2006). We accounted for this by resampling 20 individuals of both sets of eastern and western chimpanzees to equate the sample size number (Fig. 3). After one million repetitions, the nucleotide diversity of the resampled eastern chimpanzee subsets was still lower than the nucleotide diversity of the resampled western chimpanzee subsets, supporting the previous findings of the permutation test.

**Fig. 4:**
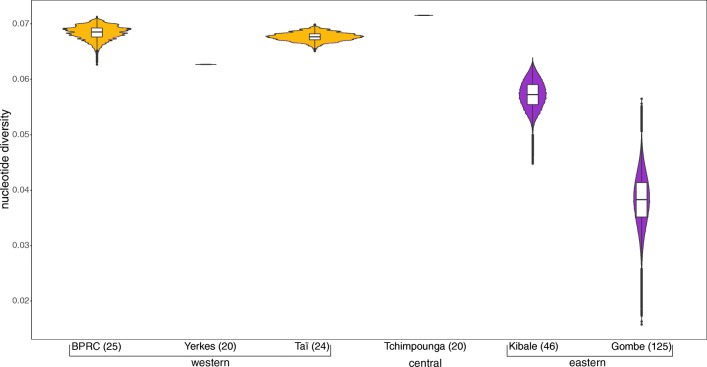
The *MHC-B* exon two nucleotide diversity comparison of our set of samples with different sets of western (golden), central (blue), and eastern chimpanzees (purple) from the literature. Sample locations and the number of individuals of each particular chimpanzee set are indicated below the plots. We resampled 20 individuals one million times from each set and calculated the nucleotide diversities. The shape of the particular plots indicates the distribution of these calculated nucleotide diversities

We next compared the exon two nucleotide diversity of our western and eastern chimpanzee samples with the exon two nucleotide diversity of two sets of captive western chimpanzees (formerly housed at BPRC, Netherlands and Yerkes, USA), one set of captive central chimpanzees (Tchimpounga Sanctuary, Republic of Congo) and one set of wild eastern chimpanzees, representing three different communities (Gombe National Park, Tanzania) (Adams et al. 2000; de Groot et al. 2010; Maibach et al. 2017; Wroblewski et al. 2015). We resampled 20 individuals from the different chimpanzee sets to account for differences in sample size number among the particular sets. The 20 central chimpanzees had the highest exon two nucleotide diversity. Interestingly, the western chimpanzees from the BPRC and Yerkes showed similar exon two nucleotide diversity compared to our set of western chimpanzees (Taï) but were slightly lower compared to the single set of central chimpanzees (Fig. 4). Although including representatives of three communities, the set of Gombe chimpanzees as well as our set of Kibale chimpanzees had the lowest exon two diversity estimates (Fig. 4).

**Fig. 5:**
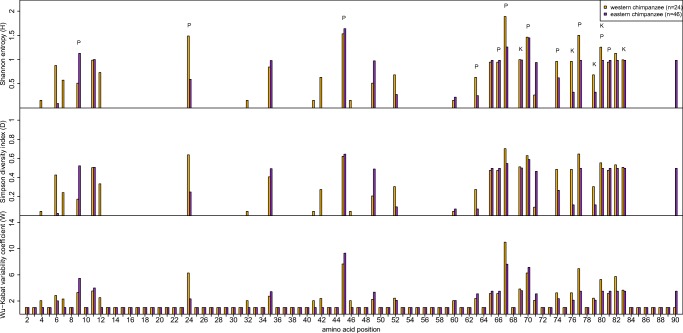
Plot of the three amino acid diversity measurements Shannon entropy, Simpson diversity index and Wu-Kabat variability coefficient for amino acid positions of the *MHC-B* exon two sequences for our two set of western (golden) and eastern (purple) chimpanzees. The number of individuals for each set as well as the positions important for the peptide binding of the MHC molecules (P) and KIR interactions (K) are indicated

We further investigated if the difference in DNA sequence diversity between our sets of western and eastern chimpanzees was also present on the amino acid level. We compared the Shannon entropy, the Simpson diversity index, and the Wu-Kabat variability coefficient between our 24 western and 46 eastern chimpanzees (Fig. 5). The three different diversity measurements indicated the same results for the individual amino acid positions except for positions 60, 63, and 70. At position 60, the Shannon entropy and the Simpson diversity index indicated a slightly lower value for our set of western chimpanzees; however, the Wu-Kabat variability coefficient indicated no difference between the two sets of chimpanzees. Furthermore, at position 63, the Shannon entropy and the Simpson diversity index indicated a higher value for our set of western chimpanzees, whereas the Wu-Kabat variability coefficient indicated a higher diversity for our set of eastern chimpanzees. In addition, at position 70, the Shannon entropy indicated no difference between our sets of western and eastern chimpanzees, in contrast to a slightly higher diversity for western chimpanzees indicated by the Simpsons diversity index or a higher diversity for eastern chimpanzees indicated by the Wu-Kabat variability coefficient. Furthermore, it is important to note that the Shannon entropy and the Simpsons diversity index showed higher values for most of the amino acid positions compared to the Wu-Kabat variability coefficient. Nevertheless, all three diversity measurements showed the same polymorphic amino acid positions for the two sets of chimpanzees, including all positions important for the peptide binding and the KIR interactions (Fig. 5). Similar analyses performed on the data from BPRC, Yerkes, Tchimpounga, and Gombe emphasized the same polymorphic positions (data not shown).

In total, our two sets of sequences contain 32 polymorphic amino acid positions. We tested for a difference in amino acid diversity between our sets of western and eastern chimpanzees by comparing the number of polymorphic amino acid positions and the diversity values at these positions. Although our set of western chimpanzees had slightly more polymorphic amino acid positions than our set of eastern chimpanzees (31 vs. 25), our set of western chimpanzees did not have significantly higher diversity at the polymorphic positions than did our set of eastern chimpanzees after Bonferroni correction (Bonferroni corrected significant threshold *p* ≤ 0.0167; Shannon entropy: mean 25.728 vs. 20.953, Wilcoxon signed-rank test, *V* = 374, *p* value = 0.041; Simpsons diversity index: mean 11.485 vs. 9.715, Wilcoxon signed-rank test, *V* = 368, *p* value = 0.053; Wu-Kabat variability coefficient: 172.099 vs. 156.905, Wilcoxon signed-rank test, *V* = 347, *p* value = 0.123).

**Fig. 6:**
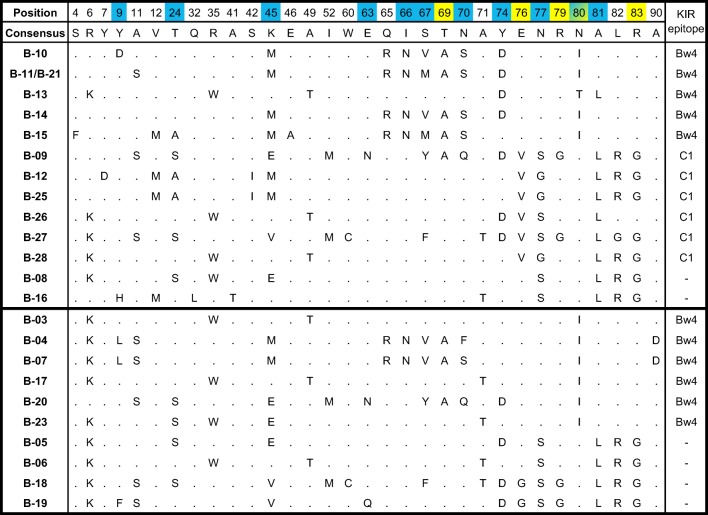
The amino acids of all 32 polymorphic positions of the MHC-B exon two molecules for our set of western (upper panel) and eastern (lower panel) chimpanzees, respectively. Within each panel, sequences are grouped by KIR epitopes and sequence designation. Similarities to the consensus sequence are indicated by dots. KIR epitopes for each sequence are indicated and defined by positions 69, 76, 79, 80, and 83 (yellow shading). Positions important for the peptide binding of the MHC-B molecules are indicated by blue shading. Position 80 is involved in the peptide binding and the interaction with KIRs

We next checked for KIR epitopes in the different MHC-B sequences of our two sets of chimpanzees. We found that for the two possible Bw4 and C1 (MHC-B-C1) KIR epitopes at the chimpanzee *MHC-B* locus, only our set of western chimpanzee sequences contained both the Bw4 and C1 epitopes, whereas our set of eastern chimpanzee sequences had only the Bw4 epitope (Tables 1 and 2; Fig. 6). The two epitopes are determined either by an arginine at position 83 (Bw4) or a valine and an asparagine at positions 76 and 80 (C1), respectively (Parham and Moffett 2013). None of the eastern chimpanzee sequences had a valine at position 76, explaining the absence of the MHC-B-C1 epitope in this set of sequences (Fig. 6). Within our set of western chimpanzees, 22 out of 24 individuals (92%) had MHC-B sequences with KIR epitopes with relatively similar frequencies between the Bw4 and the MHC-B-C1 epitope (Table 4a). Of the 46 eastern chimpanzees in our dataset, 38 individuals (83%) had MHC-B sequences with the Bw4 epitope. The number of individuals with KIR epitopes was not significantly different between our two sets of MHC-B sequences (92% vs. 83%, Fisher’s exact test, *p* = 0.8546).

**Table 4:** Frequencies of chimpanzees with the two KIR epitopes Bw4 and C1 (MHC-B-C1) compared among different chimpanzee populations/sets (a) and compared among the three subspecies (b). The subspecies comparison includes all populations/sets presented in this table and in addition few chimpanzees (14) which were not included in the population comparison because the grouping of so few individuals as several independent sets of chimpanzees would have been confusing. N represents the number of individuals

**(a)**				
**Subspecies**	**Population**	**N**	**Epitope frequency**	
			**Bw4**	**C1**
Western	Taï	24	0.58	0.54
Western	BPRC^1^	25	0.80	0.36
Western	Yerkes^2^	20	0.80	0.50
Central	Tchimpounga^2^	20	0.60	0.45
Eastern	Kibale	46	0.83	0
Eastern	Gombe^3^	125	0.48	0.10
**(b)**				
**Subspecies**	**N**	**Epitope frequency Bw4**	**Epitope frequency C1**	
Western	73	0.73	0.47	
Central	23	0.57	0.44	
Eastern	178	0.57	0.07	

^1^de Groot et al. (2010)

^2^Wroblewski et al. (2019)

^3^Wroblewski et al. (2015)

To put the results in a broader context, we next compared the frequencies of the Bw4 and the MHC-B-C1 epitopes in our samples as well as samples from other studies (Table 4; Supplementary Table 2), as previously done by Wroblewski et al. (2019), and including additional data from de Groot et al. (2010) and Wroblewski et al. (2015). With regard to Bw4, we found that the frequencies of chimpanzees with this epitope differed significantly among the different sets of chimpanzees (Fisher’s exact test of independence, *p* = 0.0005). However, subsequent pairwise analysis using a post-hoc test revealed that the frequencies of chimpanzees with the Bw4 epitope were similar among all pairwise comparisons except for the comparison of Gombe and Kibale, indicating a higher frequency of the Bw4 epitope in Kibale (post-hoc test Fisher’s exact test, *p* < 0.0001) (Tables 4a and 5). As with the Bw4 epitope, the frequency of individuals with the MHC-B-C1 epitope also differed among the different chimpanzee sets (Fisher’s exact test of independence, *p* = 0.0005). Interestingly, each non-eastern chimpanzee set (i.e., Taï, Yerkes, BPRC and Tchimpounga) had significantly higher frequencies of individuals carrying the MHC-B-C1 epitope compared to the frequency of individuals with the MHC-B-C1 epitope in Gombe and Kibale (both eastern chimpanzee sets) (post-hoc test Fisher’s exact test, *p* ≤ 0.0019; Table 5). In addition, we grouped all chimpanzees by subspecies and compared the frequencies of individuals with the Bw4 and MHC-B-C1 epitope, respectively (Table 4b). This included also some individuals (in total 14) from three different subspecies presented in Wroblewski et al. (2019) which were not included in the previous comparison because the grouping of so few individuals as several independent sets of chimpanzees would have been confusing (Supplementary Table 2). When comparing the frequencies of chimpanzees with the Bw4 epitope among the three subspecies, we found no significant difference for the Bw4 epitope (Table 4b; Fisher’s exact test of independence, *p* = 0.0648). However, as expected, based on the results for the MHC-B-C1 epitope from the previous comparison, we found higher frequencies of individuals with the MHC-B-C1 epitope in western and central chimpanzees, compared to eastern chimpanzees (Table 4b; Fisher’s exact test of independence and post-hoc Fisher’s exact tests, *p* < 0.0001), while there was no difference in MHC-B-C1 epitope frequency between western and central chimpanzees (post-hoc Fisher’s exact tests, *p* = 0.8156).

**Table 5:** Statistical results of Bw4 and C1 (MHC-B-C1) epitope frequency comparison among different chimpanzee populations. The upper right part shows the *p* values for the C1 (MHC-B-C1) epitope. The lower left part shows the *p* values for the Bw4 epitope. Significant values are indicated in italics. We corrected for multiple testing using the Bonferroni correction (adjusted significant threshold 0.05/15 = 0.0033)

Bw4\C1	BPRC	Yerkes	Taï	Tchimpounga	Gombe	Kibale
BPRC	–	0.3792	0.2563	0.5587	*0.0019*	*< 0.0001*
Yerkes	1	–	1	1	*0.0001*	*< 0.0001*
Taï	0.1284	0.1948	–	0.7626	*< 0.0001*	*< 0.0001*
Tchimpounga	0.1915	0.3008	1	–	*0.0003*	*< 0.0001*
Gombe	0.0040	0.0083	0.3812	0.3458	–	0.0376
Kibale	0.7601	1	0.0428	0.0644	*< 0.0001*	–

## Discussion

In this study, we analyzed 24 western (*P. t. verus*) and 46 eastern (*P. t. schweinfurthii*) wild chimpanzee *MHC-B* exon two sequences sampled using necropsy and fecal samples, respectively, and compared the results with those previously reported from other samples of chimpanzees. We demonstrated that, although it was challenging, we could generate *MHC-B* exon two sequences using these sample types. Although one might expect that tissue samples derived from a necropsy would provide good quality DNA, the samples were collected in field conditions at varying times after death, thus explaining the 80% PCR success rate from western chimpanzees. Similarly, although the amplicon has a relatively modest length of < 500 bp, this can be challenging to amplify from the low-quality, low concentration DNA typically obtained from feces, thus explaining the 77% PCR success rate when using fecal DNAs from eastern chimpanzees. Indeed, because the DNA is highly degraded in fecal samples and potentially contains DNA from many other organisms like bacteria, we expected a much higher failure rate than we observed (Taberlet et al. 1999; White et al. 2019). However, this relatively low failure rate reflects the intensive assessment of fecal samples quality parameters (evaluation score) prior to PCR and sequencing and our selection of 50 samples with the highest evaluation scores (White et al. 2019). Although we found only a weak correlation between the evaluation score and PCR success rate, this likely reflects range restriction, and inclusion of samples with lower evaluation scores might further validate the idea that the evaluation score predicts the samples PCR and sequencing success rate.

The presence of 13 and 10 different exon two nucleotide and amino acid sequences for our western and eastern chimpanzees, respectively, indicated a higher number of *MHC-B* alleles in our set of western chimpanzees. However, it is important to note that the *MHC-B* exon two sequences could represent several different full length *MHC-B* alleles, and therefore, the number of full length *MHC-B* alleles in these two sets of chimpanzees could differ as compared to the number of exon two sequences. The unequal distribution of the exon two frequencies in our two sets of sequences, e.g., one or two alleles with high frequencies, followed by alleles with intermediate and low frequencies, are comparable to the distribution of allele frequencies found in other studied chimpanzee and small indigenous human populations (Wroblewski et al. 2015). Interestingly, our *B-06* exon two sequence, which had the highest frequency in our set of eastern chimpanzees, had the same exon two sequence as the B*22:04 allele which had the highest frequency in Gombe eastern chimpanzees (Wroblewski et al. 2015). In addition, the *B-11* and *B-08* exon two sequences which had the highest and the second highest frequencies in our set of western chimpanzees had identical exon two sequences compared to the two alleles with the highest frequencies (B*01:01 and B*05:01) in western chimpanzees from the BPRC and Yerkes (Adams et al. 2000; de Groot et al. 2010; Wroblewski et al. 2015). The presence of the same *MHC-B* alleles in high frequencies across different chimpanzee populations implies some importance of these alleles and might indicate a subspecies-specific pattern of high-frequency *MHC-B* alleles, maybe representing the result of selection acting on these subspecies.

The higher number of exon two sequences in our set of western chimpanzees compared to our set of eastern chimpanzees hinted at a lower *MHC-B* exon two diversity in our set of eastern chimpanzees. This was supported by the lower *MHC-B* exon two nucleotide diversity in our set of eastern chimpanzees compared to our set of western chimpanzees. Interestingly, the comparison with other chimpanzee sets from different western, central, and eastern chimpanzee populations showed a similar pattern of high exon two nucleotide diversity in western chimpanzees compared to low exon two nucleotide diversity in eastern chimpanzees. This suggests that the differences in *MHC-B* exon two diversity which we found among individuals of the Taï National Park and the Kibale National Park represent a consistent difference in *MHC-B* exon two nucleotide diversity between western and eastern chimpanzees. Regarding the amino acid diversity at polymorphic sites of the *MHC-B* exon two amino acid sequences, we found no evidence of higher amino acid diversity in our set of western chimpanzees, indicating that the difference in nucleotide diversity might not cause a difference in functionality between our set of western and eastern chimpanzees. However, this assumes that a more or less equal number of amino acids at the polymorphic sites lead to an equal functionality of the particular MHC sequences, which might not be true, because of different physico-chemical properties of the individual amino acids, leading to differing abilities to interact with peptide sequences (Matsumura et al. 1992). In addition, our comparison includes all polymorphic sites and not only the sites involved in the forming of the α_1_ domain of the peptide binding groove (Bjorkman et al. 1987; Saper et al. 1991). In sum, drawing conclusions on the functional ability of the *MHC-B* exon two sequences of our two sets of chimpanzees is rather difficult and would need additional investigation of the *MHC-B* exon three sequences, which encodes for the α_2_ domain of the antigen binding site and proper MHC molecule functionality analysis like the investigation of the ability of MHC molecules to bind viral peptides using bioinformatic binding prediction tools (Hoof et al. 2009; Maibach and Vigilant 2019; Pro et al. 2014; van Deutekom et al. 2011).

The comparison of the Bw4 and C1 (MHC-B-C1) KIR epitope frequencies of the MHC-B sequences among the different chimpanzee sample sets revealed interesting findings. Previous results on the Bw4 epitope hinted at a reduced frequency of this epitope in eastern chimpanzees compared to western chimpanzees (Wroblewski et al. 2015; Wroblewski et al. 2019). However, we found in our set of Kibale eastern chimpanzees a relatively high Bw4 epitope frequency, which was comparable to the Bw4 frequencies of western and central chimpanzees and higher relative to the previous studied eastern chimpanzees from Gombe (Wroblewski et al. 2015). This indicates that differences in the frequency of the Bw4 epitope cannot be attributed solely to differences between chimpanzee subspecies, but rather suggests that two populations belonging to the same subspecies may differ substantially in their Bw4 epitope frequencies. In contrast, the MHC-B-C1 epitope frequency showed clear subspecies differences. The complete absence of this epitope at MHC-B molecules in our Kibale and other eastern chimpanzee sets (Wroblewski et al. 2019), as well as the very low frequency in eastern chimpanzees from Gombe, strongly indicates a reduced MHC-B-C1 epitope frequency in eastern chimpanzees. However, eastern chimpanzees still possess a C1 epitope at some MHC-C molecules (MHC-C-C1), as indicated by a comparison of KIR epitopes present at MHC-C molecules (Wroblewski et al. 2019). This indicates that the majority of KIR interactions of MHC-B molecules with NK-cells in eastern chimpanzees are mostly restricted to the Bw4 epitope and not to both the Bw4 and C1 epitopes as in other chimpanzee subspecies. Such changes and refinement of KIR epitope frequencies at certain MHC genes have been observed throughout the evolution of hominids, as comprehensively reviewed by Wroblewski et al. (2019). For example, like eastern chimpanzees, humans possess an extremely low frequency of the MHC-B-C1 epitope, presented only by two human MHC-B molecules, which might have been introduced into modern humans by archaic humans after complete loss of the MHC-B-C1 epitope (Abi-Rached et al. 2011; Wroblewski et al. 2019). Even further changes have been observed in the bonobos, close relatives of the chimpanzees who lack the C1 epitope on their MHC-C molecules (Wroblewski et al. 2019). In addition and more importantly, bonobos lack the specific KIR genes responsible for the interaction with C1 epitopes, indicating that even bonobos that would have the C1 epitope at some MHC-B molecules nonetheless lack the ability to interact with the C1 epitope (Rajalingam et al. 2001; Wroblewski et al. 2019). These examples demonstrate that KIR and MHC genes and their interactions constantly changed throughout hominid evolution. The differences of MHC-B-C1 epitope frequencies among the different chimpanzee subspecies show that the complex evolutionary pattern of KIR and MHC genes observed among different hominid species might also be present at the subspecies level of a single ape species.

The reduced *MHC-B* exon two nucleotide diversity in eastern chimpanzees contrasts with their higher genetic autosomal diversity, larger effective population size, and less severe genetic bottleneck compared to western chimpanzees (Fischer et al. 2006; Fischer et al. 2011; Prado-Martinez et al. 2013). This suggests that rather than demography, the differences in *MHC-B* exon two diversity and MHC-B-C1 epitope frequency might be better explained by a selective processes mediated by pathogens given the importance of this gene in immune function. The MHC diversity of both studied chimpanzee populations from the Taï and the Kibale National Parks could be affected by pathogen exposure in their past. In addition to the confrontation with several respiratory diseases which affected both studied populations, western chimpanzees from Taï also experienced severe Ebola outbreaks (Emery Thompson et al. 2018; Formenty et al. 1999; Hoffmann et al. 2017; Köndgen et al. 2008; Leendertz et al. 2017; Negrey et al. 2019; Scully et al. 2018). This potential higher pathogenic load in this population could be responsible for the selection of higher *MHC-B* exon two diversity. However, contradicting this very simplified hypothesis is the low *MHC-B* diversity in eastern Gombe chimpanzees which might also experience a high pathogenic load caused by SIV (Wroblewski et al. 2015). Another explanation for the differences in *MHC-B* exon two diversity between western and eastern chimpanzees could be the reduction of diversity in eastern chimpanzees caused by certain pathogens in their evolutionary past; however, finding a suitable pathogen candidate is too speculative.

Equally difficult to explain is the reduction of the MHC-B-C1 epitope frequency in eastern chimpanzees. Again, a selective process by pathogens could be reasonable as the interaction of KIR epitopes with KIR genes on NK cells is of similar importance for effective immune functionality as the presentation of antigens by MHC molecules to T cells. However, the reduction of the MHC-B-C1 epitope frequency of eastern chimpanzees could be simply a by-product of selection driving the increase in frequency of certain *MHC-B* alleles, which by chance are not coding for the MHC-B-C1 epitope. This is supported by the fact that the complete loss of the C1 epitope at MHC-B molecules (MHC-B-C1) might not severely reduce the ability of an individual to interact with KIRs as the C1 epitope is still present on some MHC-C molecules (MHC-C-C1) in eastern chimpanzees. In this regard, it would be very interesting to analyze if there exist differences between western and eastern chimpanzees in the presence and frequency of the particular KIR genes which allow the interaction with the C1 epitope, as was done in the comparisons between chimpanzees and bonobos (Rajalingam et al. 2001; Wroblewski et al. 2019).

In sum, in this present study, we describe differences in *MHC-B* exon two diversity and the frequency of KIR epitopes between populations of western and eastern chimpanzees which are likely not the result of differences in overall genetic diversity and population genetic history between the populations but rather represent the outcome of selective processes mediated by different pathogen pressures. To extend this work, intensive studies are needed investigating the natural pathogen environments and pathogen threats of wild living chimpanzee communities and populations which might provide insights into the selective forces causing the differences seen in MHC diversity between chimpanzee subspecies, populations, and communities.

## Electronic supplementary material

Supplementary Table 1. Primer sequences used for the amplification of the MHC-B exon two sequences (PCR I) and for the indexing of the particular sequencing libraries (PCR II). Small letters in the primer DNA sequence represent one example of the specific barcodes added to each particular sample. (XLSX 10 kb)

Supplementary Table 2. The presence or absence of the Bw4 and C1 (MHC-B-C1) KIR epitopes for all individuals used for the calculation of the KIR epitope frequencies, including data used from de Groot et al. (2010), Wroblewski et al. (2015) and Wroblewski et al. (2019). Chimpanzee subspecies, population and study from which we took the data are indicated. (XLSX 16 kb)

## Abbreviations

MHC: Major histocompatibility complex
KIR: Killer cell immunoglobulin-like receptors
NK cells: Natural killer cells
BPRC: Biomedical Primate Research Centre
